# An evaluation of spraying as a delivery method for human mesenchymal stem cells suspended in low-methyl pectin solutions

**DOI:** 10.1186/s13287-025-04331-4

**Published:** 2025-05-16

**Authors:** Ami Nash, I-Ning Lee, Graeme Fox, James Phillips, Lisa J White, Maria Marlow

**Affiliations:** 1 School of Pharmacy, University of Nottingham, Nottingham, NG7 2RD UK; 2 Deep Seq, Centre for Genetics and Genomics, University of Nottingham, Queen’s Medical Centre, Nottingham, NG7 2UH UK; 3 Department of Pharmacology, School of Pharmacy, University College London, London, WC1N 1AX UK

**Keywords:** Human mesenchymal stem cells, Spraying, Pectin

## Abstract

**Background:**

Mesenchymal stem cells have shown promise in many areas of regenerative medicine due to the anti-inflammatory and pro-regenerative effects of the secreted factors. However, successful delivery remains problematic, particularly for delivery to areas such as the brain. Spray delivery is a method investigated in wound care and lung injury, which may be applicable for brain delivery to patients already requiring surgery. To retain therapeutic mesenchymal stem cells at the delivery site, biomaterials can be employed; pectin is a biocompatible, sprayable, and mucoadhesive material, which could prove suitable for spray delivery of cells for therapeutic uses.

**Methods:**

The biocompatibility of four grades of low-methyl pectin gelled by addition of calcium was assessed using SH-SY5Y cells. After, mesenchymal stem cells were suspended within the four different grades of low-methyl pectin solutions and sprayed using a syringe-driven spray device. The suitability was then assessed by cell viability testing, flow cytometry to test for surface markers, and differential gene expression studies to understand the effects of both the pectin and the spraying process on the gene expression of the cells.

**Results:**

All four grades of low-methyl pectin were biocompatible with SH-SY5Y cells. The syringe-driven spray device delivered human mesenchymal stem cells to well plates with high viability, and suspending these cells in pectin solutions for spraying did not negatively affect the viability. The grade of pectin named CU-701 was the best grade based on results of the flow cytometry, whereby the surface marker expression was not altered from the control cells. The RNA sequencing showing the differential expression showed that the process of spraying the cells did not alter gene expression compared to the control, however the pectin, and the presence of calcium used to induce gelation of the pectin, did lead to altered gene expression in cells.

**Conclusion:**

Spraying is a suitable delivery method for the mesenchymal stem cells, showing no detrimental effect on the cells. Pectin shows little effect on the viability of the cells, however the use of calcium to gel the pectin appears to affect the expression of several genes.

**Supplementary Information:**

The online version contains supplementary material available at 10.1186/s13287-025-04331-4.

## Background

Mesenchymal stem cells (MSCs) have received significant attention in regenerative medicine due to their therapeutic potential in treating various diseases and tissue injuries [1]. Their ability to modulate immune responses [2], promote tissue regeneration [3], and secrete trophic factors [4] makes them valuable candidates for cell-based therapies. However, the success of MSC-based therapies depends on the efficient delivery of these cells to target tissues or organs. Delivery methods such as direct injection or systemic infusion, often encounter limitations such as poor cell retention, limited targeting, and potential safety concerns [5, 6]. This is particularly poor when considering their use for the treatment of brain injuries. It has been estimated that < 1% of the delivered cells will make it to the brain [7], with the majority becoming trapped within small pulmonary capillaries [6].

Local delivery to the brain is an option to overcome the limitations of systemic delivery, however this is most viable for patients already receiving surgery for their brain injury. It is estimated approximately 10% of severely injured patients will require some form of surgery [8], therefore local delivery lends itself as an additive therapy for these patients. Delivering MSCs locally has previously shown improved efficacy over intravenous delivery in brain injury models, with the improvement being attributed to increased cell number in the brain or cells being localised to the site of damage [9]. Local delivery by spraying is one possible local delivery method; it involves the generation of finely dispersed droplets containing MSCs, which can be directly administered to target tissues or organs. This approach offers several possible advantages, including uniform distribution of cells within the target area, and enhanced targeting capabilities, which have been investigated for use in burn wound care [10–12], as well as delivery of cells for lung injury [13, 14].

Following delivery, retaining therapeutic cells at the site of injury by inclusion of a hydrogel may enhance therapeutic effects. In stroke models, hydrogels have shown promising results in lesion repair when used in conjunction with therapeutic cells [15, 16]. Fibrin has been used to deliver cells to the injury site in rat models of TBI with enhanced recovery observed when compared to no fibrin [9].

Hydrogel selection for use with spraying must be carefully considered, as materials with too high viscosity will increase cell death [17]. Materials that have a low viscosity and form gels in situ may be suitable for use for cell delivery by spraying and to retain cells at the site of injury. Pectin, specifically low-methyl (LM) pectin with < 50% methylation of the carboxyl groups of the sugar backbone, is one such material. Pectin is a solution until it forms a hydrogel with the addition of Ca^2+^ [18]. Calcium is found endogenously in the brain, with an extracellular concentration of ~ 1 mM [19]. In combination with the strong mucoadhesive properties of pectin [20], this may be sufficient to retain cells.

Biocompatibility of pectin has been demonstrated in humans, for use as a plasma expander [21]. More recently, it has demonstrated biocompatibility when used as a material for chemotherapeutic nanoparticle delivery to rats’ brains [22], and for the encapsulation of MC3T3-E1 preosteoblast cells [23], L929 mouse fibroblasts [24], and hMSCs [25]. Pectin has also demonstrated some anti-inflammatory properties [26], which could aid in the treatment of brain injury. In addition to the degree of methylation (DM), pectin can also be amidated on the carboxyl groups on the sugar backbone, and the extent of amidation is referred to as the degree of amidation (DA). The DM and DA affect the gelation properties and the sensitivity to gelation by Ca^2+^, with lower DM and higher DA forming stiffer gels at lower Ca^2+^ concentrations [27].

Many previous spraying studies have utilised airbrush systems, using high pressure air to generate the cell-containing droplets [12–14, 28]. The airbrush delivery method has advantages in delivery for burn wound care for the rapid coverage of a large surface area. However, the high pressure often results in the death of a large proportion of the cells [29]. Therefore, as large surface area is not required for applications in brain injury, a syringe-driven spray device may be more appropriate.

The aim of this work was to investigate the suitability of a syringe-driven spray device, and pectin, for use in delivering hMSCs. Previous work using pectin with cells has largely focused on the LM pectin grade CU-701 [30, 25], therefore this grade, along with three others differing in DM and DA, were investigated for the delivery by hMSCs by spraying to understand if the differing DM and DA affects the cells. The viability of the cells was tested, as well as the surface markers of the hMSCs following spraying in pectin solutions. One grade was then selected for transcriptomic analysis, based on the results of the viability testing and flow cytometry results. This was carried out to further understand the effect of spraying and the pectin on the hMSCs used.

## Materials and methods

Four grades of pectin were investigated for their suitability in the spray delivery of hMSCs. The different grades were all LM pectin with different DM and DA.

### Pectin Preparation

Pectin was purified prior to use, using a method adapted from a previously published protocol [25]. Briefly, 2% w/v activated charcoal (Norit SX Plus Cat, Sigma) was added to 1% w/v pectin solutions in deionised H_2_O (pH ~ 6.0) and stirred on a magnetic stirrer at room temperature for 1 h. Solutions were spun at 19,500 xg for 45 min. The supernatant was removed, and this was spun again for a further 30 min at 19,500 xg. The resultant product was filtered through 0.22 μm (LM-102, LM-104 and CU-L) or 0.45 μm (CU-701) syringe filters before being lyophilised (Biopharm freeze drier) for 24–48 h depending on batch size for storage at -20 °C in order to prevent microbial growth. Pectin gelation was carried out using filter sterilised CaCl_2 (aq)_ solutions at 10% v/v of the pectin solution (e.g., 10 µL CaCl_2_ with 100 µL pectin used).

### Cell culture

SH-SY5Y human neuroblastoma cells were maintained in Minimum Essential Media (MEM): Ham’s F12 Nutrient Mix (1:1) (Gibco) supplemented with 10% v/v FBS, 1% v/v non-essential amino acids (Gibco), and 1% v/v antibiotic/antimycotic. Cells were incubated at 37 °C with 5% CO_2_ in T75/T225 tissue culture treated flasks. Cells were passaged when they reached approximately 80–90% confluency.

A commercial source of human mesenchymal stem cells extracted from bone marrow (PT-2501 Lonza (Table 2)) were maintained in MSCGM™ Mesenchymal Stem Cell Growth Medium BulletKit™ from Lonza (PT-3001), containing foetal bovine serum, L-Glutamine, and gentamycin (GA-1000) as supplied. Cells were incubated at 37 °C with 5% CO_2_ in T75 tissue culture treated flasks. Cells were passaged when they reached approximately 80% confluency. For experiments, cells from three separate donors were used. Cells were used between passage 3 and 5.

### Viability testing

Extraction and direct cytotoxicity tests were carried out using the international standard for biological evaluation of medical devices, ISO 10993-5 and − 12 ([ISO] [31], [ISO] [31],, as a guide. The ISO standard states that testing should be carried out in established cell lines, hence the choice of SH-SY5Y cells. The lengths of incubations used for the test, and the viability testing methods were also listed in the ISO standard. The extraction and direct testing parameters were adapted for use with hydrogels, and volume ratios were calculated according to Sect. 10.3.3. Table 1 (found in ISO 10993-12 [31]), irregularly shaped solids, giving a suggested extraction ratio of 0.2 g/mL pectin hydrogel in extraction media.

**Table 1:** Supplier for each grade of pectin used, the DM, the DA, and the pH of pectin dissolved in water at 1% w/v concentration

Supplier	Grade	Abbreviation	DM	DA	pH
Herbstreith and Fox	Classic CU-701	CU-701	37%	0%	3.4
	Classic CU-L 094/19	CU-L	28%	0%	~ 4
CP Kelco	GENU LM 102 AS	LM-102	30%	19%	4–5
	GENU LM 104 AS	LM-104	27%	20%	4–5

SH-SY5Y cells were seeded at 25,000 cells/cm^2^ 24-hours before samples were added for testing. Pectin samples were then added to SH-SY5Y cells and left on for 24-hours before testing metabolic activity and cytotoxicity using the prestoBlue assay and LDH assay respectively. Media containing 10% v/v triton-X 100, and media containing no pectin were used as the positive and negative controls.

The PrestoBlue assay (Invitrogen), CyQUANT™ LDH Cytotoxicity Assay (Invitrogen), and LIVE/DEAD Viability/Cytotoxicity Kit for Mammalian Cells (Invitrogen) were carried out according to manufacturer’s protocols. After the 24-hour test period, media was removed and 50 µL used for the LDH cytotoxicity assay. To this, 50 µL of the Reaction Mixture was added, incubated at room temperature for 30 min, followed by 50 µL of Stop Solution. Absorbance was read at 490 nm and 680 nm. On the remaining cells, media was replaced with media containing 10% v/v prestoBlue. Plates were incubated for 2 h at 37 °C. Media was transferred in triplicate to a black 96-well plate and fluorescence read– excitation 550 nm, emission 590 nm. LIVE/DEAD stain was prepared by adding calcein AM (1/5000), and ethidium homodimer-1 (1/1000) to 1xPBS. NucBlue™ Live ReadyProbes™ Reagent (Hoechst 33342) (ThermoFisher) was also added at a concentration of 1 drop per mL, prior to imaging by fluorescent microscopy.

### Cell spraying

The Mucosal Atomisation Device (Teleflex) was selected as the syringe-driven spray device. The device was repurposed for this study from being a nasal device; to investigate its use for delivery directly onto tissue surfaces, ultimately the surface of the injured brain during surgery. As such, the nasal shield was removed, and the device was attached to a 1 mL syringe (Henke Sass Wolf) for cell spraying experiments. The device was sterilised using 70% IMS solutions and rinsed prior to use. To spray hMSCs, cells were detached using Accutase (Gibco) for a maximum of 15-minutes or until the cells were visibly detached, resuspended in a minimal volume, and counted. This cell suspension was then used to prepare the 1% w/v pectin solutions with the desired densities; final cell density was 1 × 10^5^ cells/mL, unless otherwise stated. Cells were sprayed from the top edge of the well plate to maintain height consistency, at a rate of ~ 4 s per mL into wells containing CaCl_2_ solutions at stated concentrations.

### Cell syringe

To serve as a control for the spraying process during experiments, the spray device was removed from the syringe, and cells were seeded by syringe. The cells were prepared for the syringe seeding as described above (cell spraying).

### Flow cytometry

The human MSC analysis kit from BD Biosciences (Cat. No. 562245) containing fluorophore-labelled antibodies CD90 (FITC), CD105 (PerCP-Cy5.5), CD73 (APC), for positive identification, and CD34, CD11b, CD19, CD45, and HLA-DR (PE) for negative identification. Cells were harvested from pectin gels 24 h after seeding using EDTA to chelate the Ca^2+^ and a series of 1xPBS wash steps. Cells were then resuspended in BD Pharmingen™ Stain Buffer (Cat. No. 554656). The CytoFLEX S flow cytometer was set up to record 10,000 events within the gated parameters (single cell events), or run for 90 s, whichever came sooner. The Kaluza analysis software (Beckman Coulter) was used. The following gating parameters were set against isotype control samples: CD90 FITC: >0.8%, < 1%. CD105 PerCP-Cy5.5: >4.5%, < 5%. APC CD73: >0.8%, < 1%. PE negative cocktail: >0.8%, < 1%.

### Transcriptome analysis

Cells were extracted from pectin gels after 24 hours using EDTA to chelate the Ca^2+^ and a series of 1xPBS wash steps. RNA extraction was carried out using the Qiagen RNeasy^®^ mini kit following the manufacturers protocol. The Qubit Fluorometer and Qubit RNA BR assay kit (ThermoFisher Scientific, Q10211) were used to determine RNA concentration. The Agilent TapeStation 4200 and Agilent RNA ScreenTape Assay Kit (Agilent, 5067–5576 and 5067–5577) was used to determine the RNA integrity number. 100 ng of extracted RNA was then used to make a cDNA library for sequencing using the QuantSeq 3’ mRNA-Seq library prep kit for Illumina (FWD) (Lexogen, 015.96). These were indexed using the Lexogen i7 6nt Index Set (Lexogen, P04496). 14 cycles of PCR were carried out for all samples.

Libraries were quantified using the Qubit Fluorometer and the Qubit dsDNA HS Kit (ThermoFisher Scientific, Q32854). Library fragment-length distributions were analysed using the Agilent TapeStation 4200 and the Agilent High Sensitivity D1000 ScreenTape Assay (Agilent, 5067–5584 and 5067–5585). Libraries were pooled in equimolar amounts and final library quantification was performed using the KAPA Library Quantification Kit for Illumina (Roche, KK4824). Library pool sequenced on the Illumina NextSeq 500 platform, on a NextSeq 500/550 Mid Output v2.5 150 cycle kit (Illumina, 20024904), to generate approximately 5 million 75 bp single end reads per sample. Data was converted to FastQ format using ‘bcl2fastq2’, part of the Illumina Secondary Analysis Software (v1.37.0). All data analysis was performed using the Lexogen QuantSeq workflow; a pipeline developed specifically for data generated from the QuantSeq 3’ mRNA-Seq library prep kit for Illumina (FWD). The workflow is as follows: During the Lexogen QuantSeq workflow, raw reads were trimmed of polyA tails and Illumina NextSeq adapters using Cutadapt [32]. Resulting fragments of < 20 bp were removed. Trimmed reads were aligned to the human reference genome (Homo sapiens GRCh38_emsembl_release_107_ERCC_SIRV) using the STAR aligner [33]. Aligned reads were passed to ‘featureCounts’ [34] to classify and count each alignment against the reference, using the appropriate annotation data. For differential gene analysis, comparisons were drawn between the various contrasts specified above and analysed using the ‘R’ package ‘DESeq2’ [35]. Each contrast was assessed for significantly differentially expressed genes (adjusted p-value < 0.1).

### Statistical testing

One-way ANOVA with a Dunnett’s post-test was carried out to determine statistical significance in viability assays with multiple test groups to compare to a live control. Where sprayed conditions were compared to a control condition delivered by syringe, statistical significance was determined using a student’s paired t-test.

## Results

Initially, to evaluate the biocompatibility of the four grades of pectin, the human neuroblastoma SHSY5Y cell line was used. Following this, hMSCs were used to determine the suitability of the spray device with pectin solutions to deliver the cells.

### Pectin biocompatibility testing

**Fig. 1:**
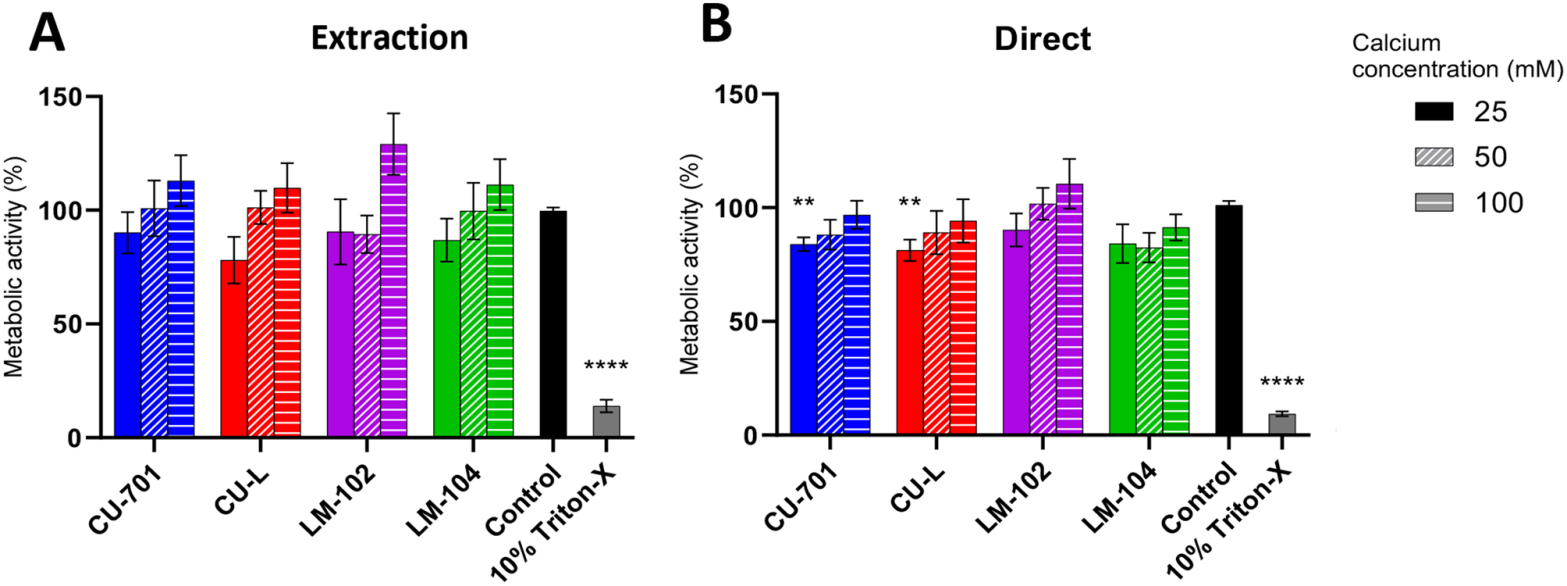
Metabolic activity of SH-SY5Y cells after cytotoxicity testing, determined by PrestoBlue assay. (**A**) media pre-incubated with pectin hydrogels formed with 25, 50 or 100 mM CaCl_2_ for 24 h was placed on cells for 24 h. (**B**) pectin hydrogels formed using 1% w/v pectin (four grades) in 1xPBS, gelled using 25, 50 or 100 mM CaCl_2_ were placed directly on cells for 24 h. Cells treated with 10% Triton-X were the positive control. The metabolic activity is calculated as a percentage of the media-only control. Three technical replicates were carried out on three separate occasions. Error bars show SEM. One-way ANOVA with a Dunnett’s post-test was carried out to determine statistical significance between tested hydrogels and an untreated control. ** shows *P* < 0.01, **** shows *P* < 0.0001

Figure 1A shows there were no statistically significant differences in the metabolic activity of the control cells and those in contact with media pre-incubated with pectin (extraction) from any of the four grades or concentrations of calcium. In direct contact testing (Fig. 1B), cells in contact with pectin gels formed using CU-701 or CU-L pectin grades (non-amidated) with 25 mM CaCl_2_ showed statistically significant lower metabolic activity than the control cells, where metabolic activity was reduced to ~ 80% compared to the control. Of all grades of pectin tested, the highest metabolic activity was observed when 100 mM CaCl_2_ was used. Having confirmed the biocompatibility of all the four pectin grades based on viability > 70%, we then progressed to spraying the hMSCs cells firstly without pectin and then in pectin solutions.

### Spraying biocompatibility testing

**Fig. 2:**
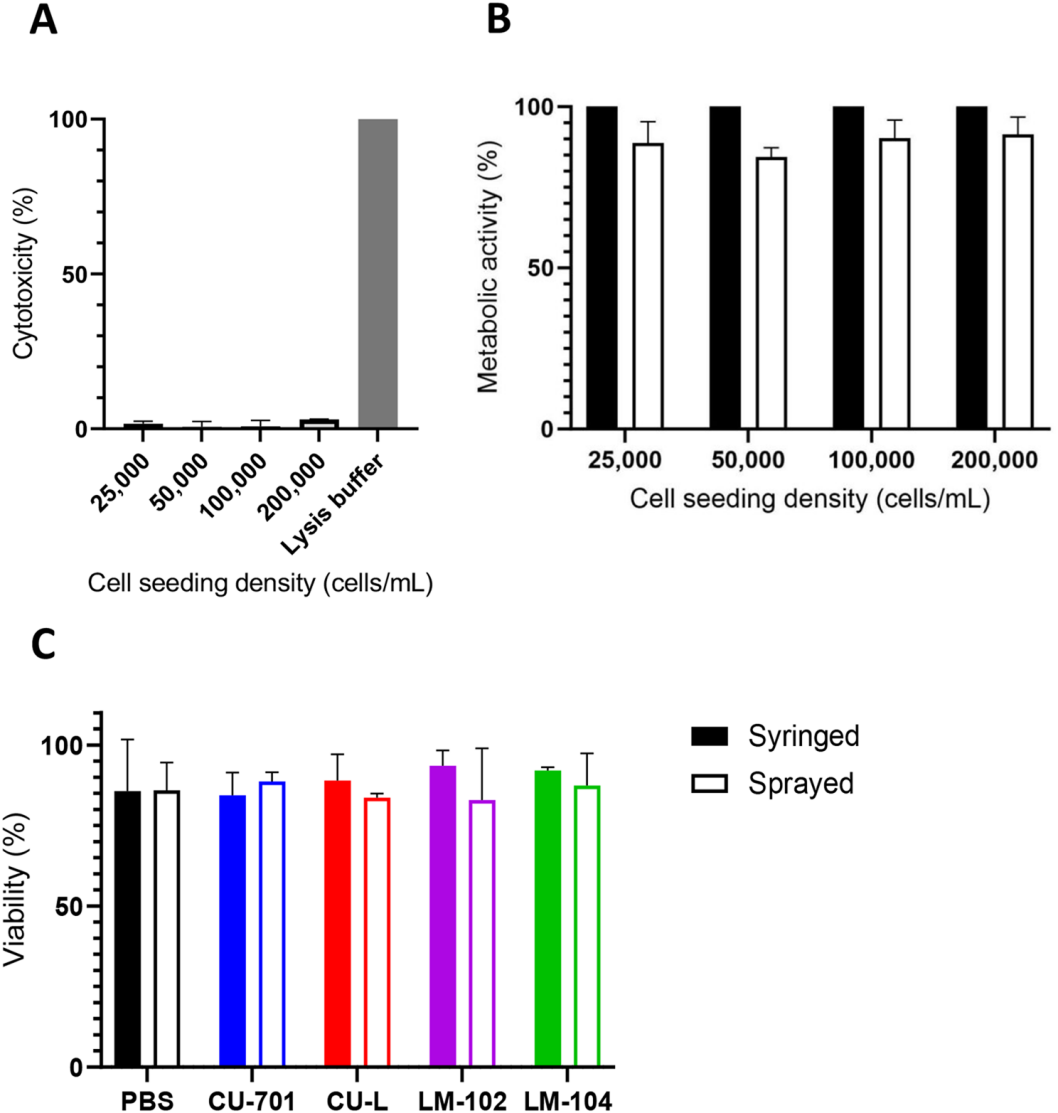
– hMSC survival after spray delivery. (**A**) Lactate dehydrogenase (LDH) assay of the sprayed hMSCs at different cell densities (2.5 × 10^4^, 5 × 10^4^, 1 × 10^5^, and 2 × 10^5^ cells/mL) showed very little cytotoxicity. (**B**) PretoBlue assay of the sprayed hMSCs at different cell densities (2.5 × 10^4^, 5 × 10^4^, 1 × 10^5^, and 2 × 10^5^ cells/mL) showed no significant difference between sprayed groups and control groups seeded by syringe. (**C**) Cell survival determined by calcien AM (live) and ethidium homodimer-1 (dead) staining of hMSCs 24 h after spraying in 1xPBS or 1% w/v pectin solutions. No difference was observed between the pectin and 1xPBS groups or the sprayed compared to control groups seeded by syringe. Three technical replicates were carried out on hMSCs from three separate donors. Error bars show SEM

The results of the LDH assay for hMSCs cells 24 h after spraying in Fig. 2A indicates the spraying process does not result in cell death. The prestoBlue assay (Fig. 2B) indicates a slight reduction in the metabolic activity of the cells 24 h after spraying (maximum 15% in the 50,000 cells/mL density), however this difference is not statistically significant. hMSCs were then sprayed at a density of 100,000 cells/mL in PBS or 1% pectin solutions (gelled in the plate using 100 mM CaCl_2_ solutions). Live/Dead staining 24-hours later (Fig. 2C) showed hMSCs seeded in all four grades of pectin, either by syringe or spray, demonstrated very similar viability to cells seeded in PBS. There were no statistically significant differences between the conditions. Hence, following confirmation of viability, flow cytometry analysis was carried out on the hMSCs to determine whether the spraying process or the four different pectin grades affected the expression of the surface markers.

### hMSC surface markers

**Fig. 3:**
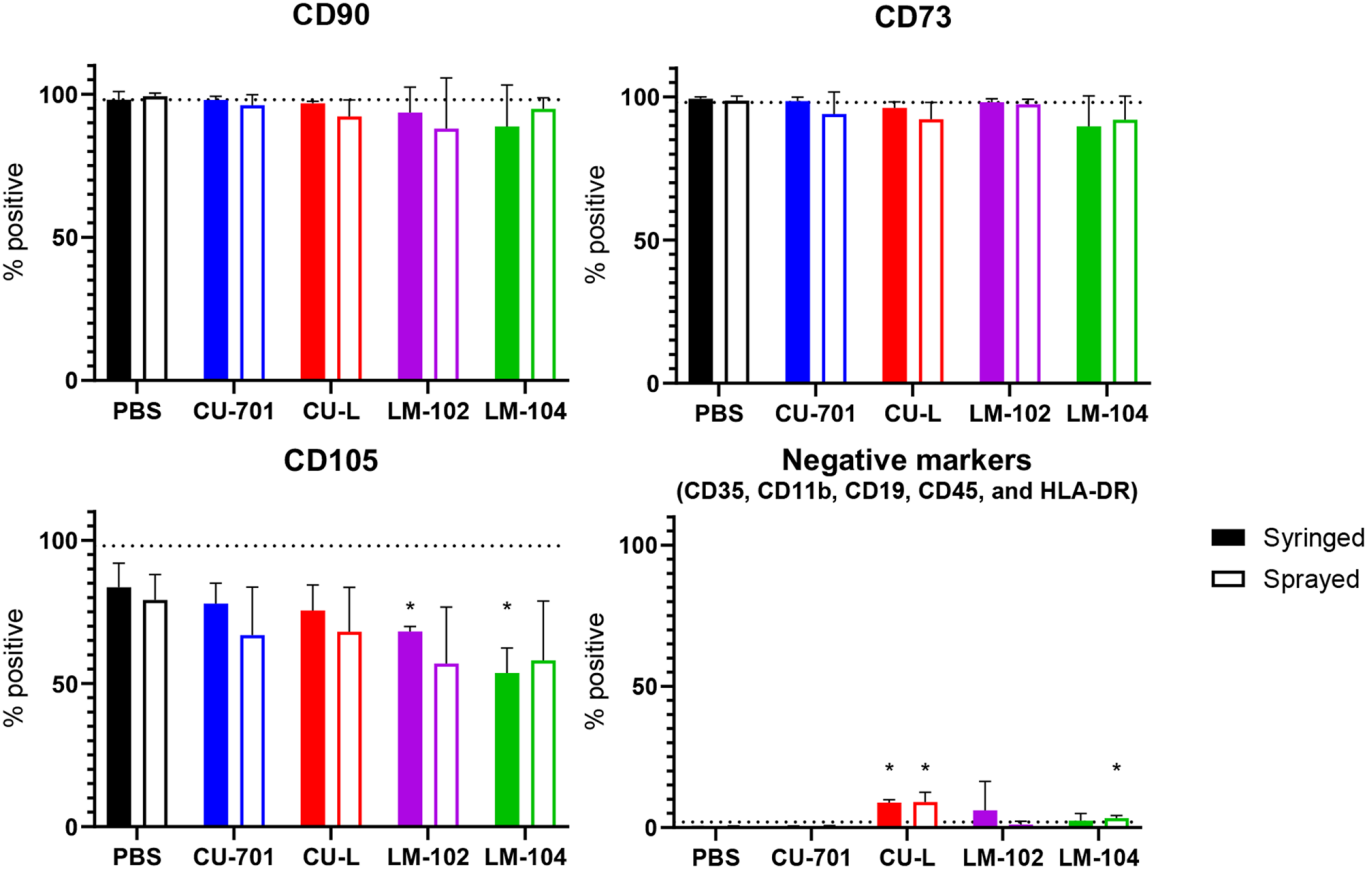
hMSC surface marker analysis carried out by flow cytometry. hMSC populations should be > 95% positive for CD90, CD105 and CD73, and < 2% positive for CD34, CD11b, CD19, CD45, and HLA-DR. Experiments were carried out on cells from three donors. Error bars show SEM, student’s paired T-test was carried out between PBS syringe condition and all other test conditions, * indicated statistical significance *P* < 0.05

Figure 3 shows the results of the flow cytometry analysis of the CD90, CD73, CD105, and pooled analysis of CD34, CD11b, CD19, CD45, and HLA-DR. The cells delivered to the plates in PBS either by spray device or syringe seeding, met the surface marker criteria to be classed as an MSC [36] for CD90, CD73, and the negative pool, however all samples analysed for CD105 were below the 95% threshold. Of the hMSCs delivered in the pectin solutions either by spray device or syringe seeding, the CU-701 grade of pectin was the only grade of pectin to demonstrate no statistically significant differences to the cells delivered in 1xPBS for all cell surface markers tested. In each of the other grades of pectin (CU-L, LM-102, and LM-104), there was a population of cells positive for the pool of markers for which cells should be negative (CD34, CD11b, CD19, CD45, and HLA-DR). Since the CU-701 grade was the only pectin grade not to have statistically significant changes in surface marker expression, this was selected for progression to hMSCs differentiation studies (Figure S4). hMSCs tested appeared to retain their potential to differentiate into osteogenic, adipogenic, and chondrogenic cells. The chondrogenic differentiation spheroids in pectin candidates are very small, which appears to be due to the presence of residual pectin limiting spheroid formation. To further understand the effect of spraying and the presence of pectin on the hMSCs, transcriptome analysis was carried out.

### RNAseq

RNA-sequencing and differential gene expression was carried out using the CU-701 pectin as this was the only grade of pectin not to show a statistically significant change in expression of surface markers.

**Fig. 4:**
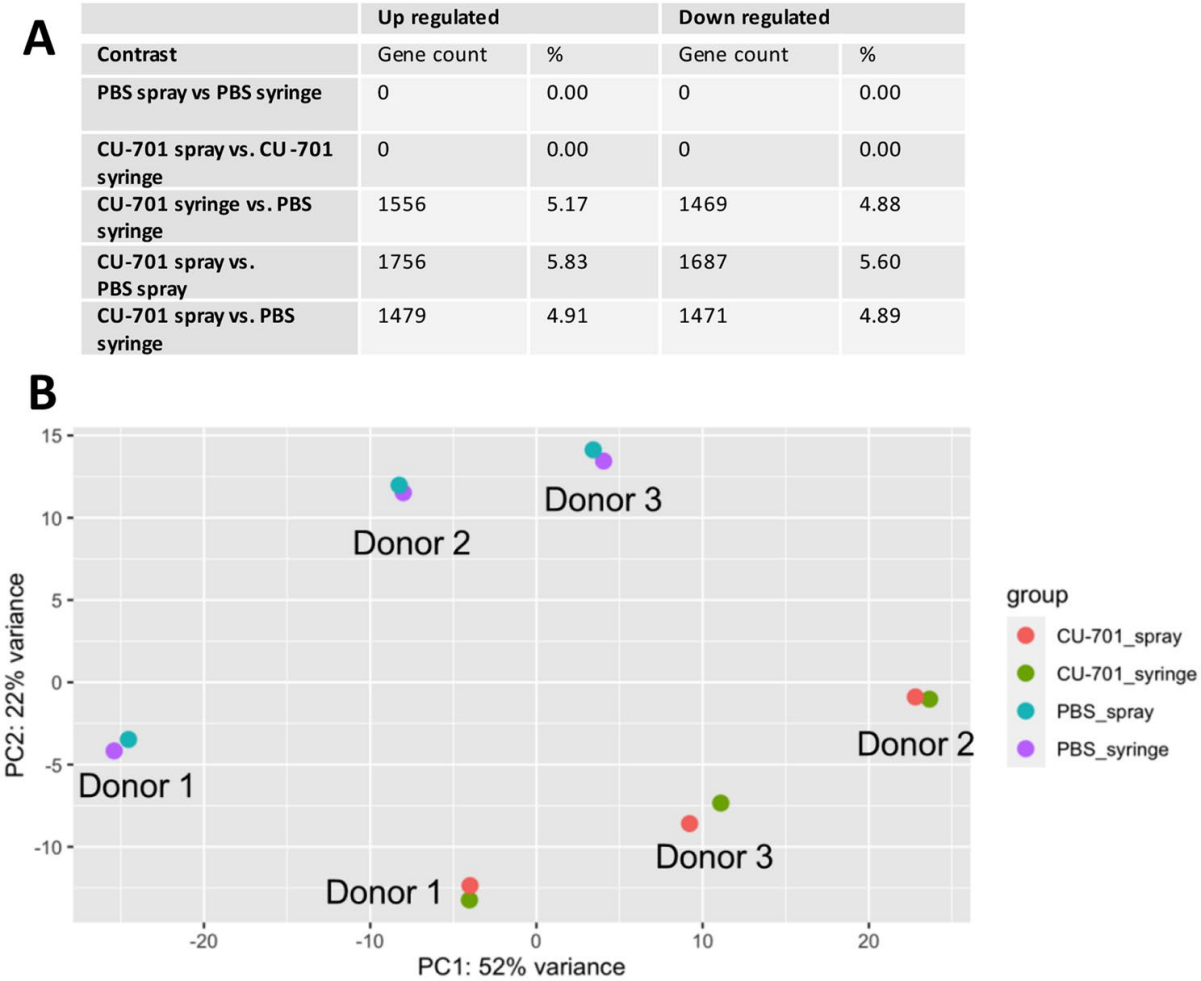
Differential gene expression analysis of hMSCs. Experiments were carried out on hMSCs from three separate donors. (**A**) Summary of the number of differentially expressed genes between the four conditions; cells seeded by syringe in PBS or CU-701 pectin, and cells seeded by spray in PBS or CU-701 pectin. (**B**) Principal component analysis (PCA) coloured by condition, with points relating to cells from each cell donor labelled

The summary of the RNAseq data shown in Fig. 4 highlights the process of spraying when compared to using a syringe to seed cells does not alter gene expression within the hMSCs used. This is shown both when comparing cells seeded by spray to those seeded by syringe in PBS (PBS spray vs. PBS syringe), and when comparing sprayed to syringed cells in pectin (CU-701 spray vs. CU-701 syringe). However, the use of pectin compared to PBS for both delivery by syringe and spray results in the up- and down-regulation of genes. This change in gene expression is also shown in the PCA plot, which additionally highlights the variation in gene expression resulting from hMSCs from three different donors. Points on the plot are separated based on the delivery material, or the donor from which the cells were obtained, but not due to the method of delivery.

**Fig. 5:**
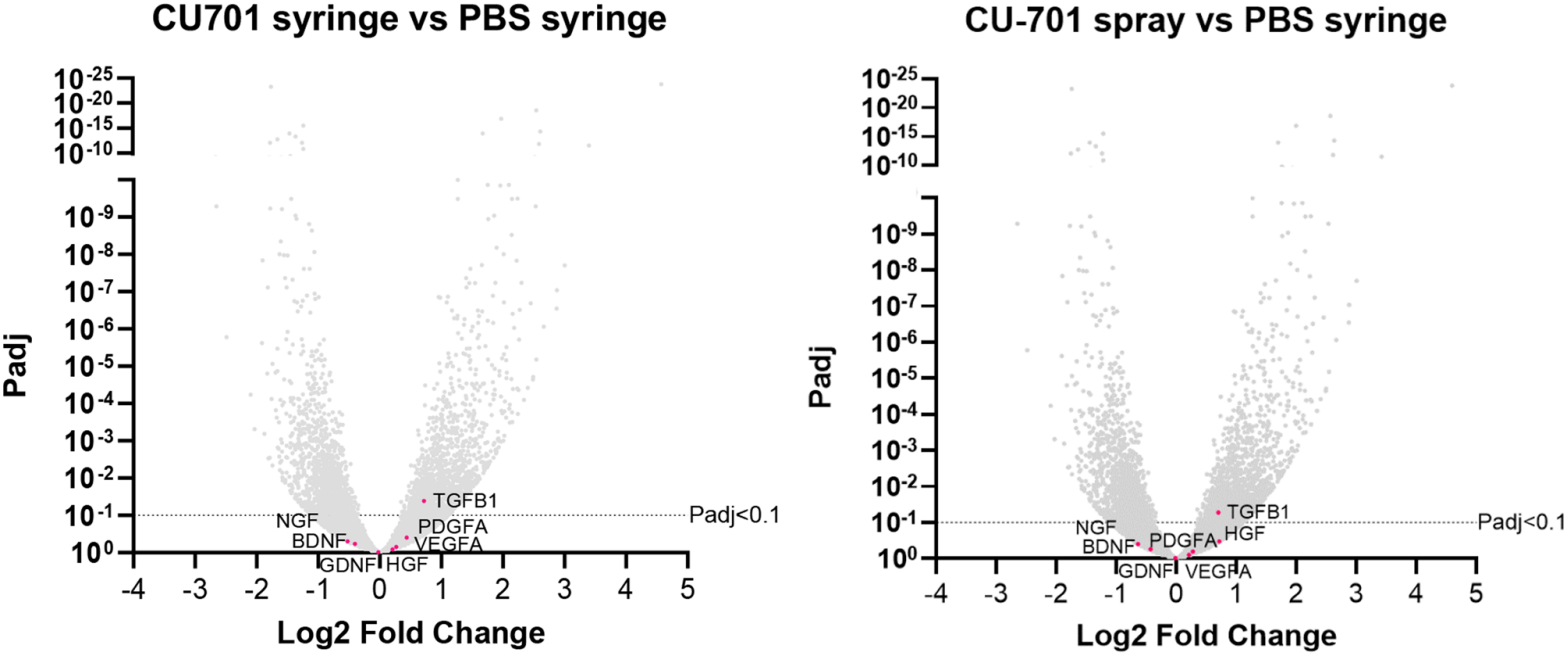
Volcano plots of the differential gene expression analysis of the CU-701 syringe vs. PBS syringe (seeded by syringe), and CU-701 spray (seeded by spray) vs. PBS syringe contrasts. Genes highlighted in pink (labelled) have been selected from literature [37, 38, 4] as important for the anti-inflammatory and pro-regenerative properties of hMSCs. An adjusted P value (Padj) of > 0.1 was deemed statistically significant

Volcano plots in Fig. 5 highlight the change in gene expression of those genes identified as important to the anti-inflammatory and pro-regenerative effects of hMSCs (Table 2) when used therapeutically. The expression of *TGFB1* is significantly upregulated in both contrasts, but the expression of all other selected genes is not significantly altered.

**Table 2:** Donor number, age, and sex of donors of hMSCs

Donor number:	Age:	Sex:
26,000	20	Male
27,519	21	Female
33,357	28	Male

## Discussion

The aim of this paper was to assess the suitability of both a syringe-based spray device, and pectin, for the spray delivery of hMSCs. Firstly, in vitro SH-SY5Y cell metabolic data in this study demonstrates the biocompatibility of a variety of pectin grades for use with cells, further demonstrated by survival of hMSCs, supporting previous studies. Historical studies have demonstrated the biocompatibility of pectin intravenously in humans (Hartman, 1943), and more recently in the brains of rat [22]. Combined, these indicate pectin may be suitable as a delivery material for hMSCs.

Figure 1 indicates a trend towards a lower metabolic activity of cells with a lower calcium concentration used to form the hydrogels. Previously, this has been hypothesised to be due to the sequestration of calcium from the culture medium [22], due to the charge interactions between calcium and carboxyl groups on the pectin. As a result, the highest concentration of CaCl_2_ (100 mM) was selected for further investigation. In addition to the higher viability observed, the gels showed a longer degradation time, which was more suitable for the in vitro studies being undertaken.

Having demonstrated the biocompatibility of pectin in vitro, our focus was then on delivering hMSCs using the spray device both with and without pectin. When using the mucosal atomisation device sprayer, cells demonstrated high viability at all densities tested, supporting previous studies using different spray devices [28, 39]. When cells were delivered by spraying in pectin solutions, the viability was not affected compared to the controls. Increased viscosity of the cell solution has been correlated with a reduction in the viability of delivered cells, with viscosity measurements of 25 mPa∙s in PEG-based delivery solutions [17] and 15 kPa∙s in dextran solutions [40] leading to a significant reduction in viability due to increased shear stress upon delivery. However, even the most viscous pectin grade used here (CU-701 with viscosity of 11.1 mPa∙s at 10,000 1/s shear rate, Figure S1) demonstrated high viability. CU-701 pectin had the highest DM, and the highest viscosity, with the other three pectin grades decreasing in viscosity as the DM decreases. This supports previous work showing the higher DM in pectin, the higher the viscosity [41].

Following on from the confirmation of hMSC viability after spraying, flow cytometry was used to confirm cell surface markers characteristic of hMSCs [36]. The CD90 and CD73 flow cytometry markers indicate that the population of hMSCs used from all three donors demonstrated appropriate expression (> 95%), however, CD105 demonstrated a reduced level of expression compared to pre-defined thresholds [36]. The overlay of the histogram plots of each sample demonstrates a homogeneous population of cells with the peak shifted to the right (Figure S2) when compared to cells stained with iso-control antibodies, however the shift is less pronounced than for the CD90 and CD73 antibodies. This either indicates a weaker binding or lower fluorescence of the antibody, or the cells have a low expression of CD105 (Figure S2). Reduced expression of CD105 has been linked to culture in serum free media, with expression levels down to approximately 50% when serum free media was used [42]. This could explain the result herein as the hMSCs tested here were cultured for 24 h in half media (as media is added in a 1:1 volume ratio to 1xPBS/pectin). Nonetheless, all three donor cell populations sprayed with 4 grades of pectin did not display reduction in CD105, in addition to CD90 and CD73, compared to PBS as control indicating spraying process of hMSCs with pectin did not affect their phenotype.

CU-701 pectin was selected for transcriptome analysis studies to further understand the effect of the process of spraying hMSCs in pectin solutions. This grade of pectin demonstrated the lowest population of cells that displayed negative markers by flow cytometry. Investigations by Curran et al. into the effects of specific functional groups demonstrated that methyl groups maintain MSC phenotype [43], which may explain the flow cytometry result as CU-701 had the highest DM of the four pectin grades.

hMSCs delivered using the spray device compared to the syringe did not have any significantly up or down regulated genes. This includes genes relating to apoptosis and senescence, indicating that the mechanical stress of spraying did not induce apoptosis or senescence of the hMSCs studied. The presence of pectin caused a change in gene expression compared to hMSCs delivered in PBS. Differences in stiffness of alginate gels (3 kPa vs. 30 kPa) has previously been shown to lead to differences in gene expression of MSCs [44]. This indicates some changes in gene expression could be due to the stiffness of the substrate they are grown within; hMSCs in pectin are in a soft gel for 24-hours before analysis, compared to those on tissue-culture plastic. Genes that appear in both the top 20 up- or down-regulated genes for CU-701 syringe versus PBS syringe, and CU-701 spray versus PBS spray contrasts are *C2CD4A*,* PDK4*,* IL24*,* IGFBP5*, and *BMF*. The expression of these genes is linked to calcium [45–48], and is therefore likely linked to the presence of calcium within the pectin hydrogels. This is supported by the gene ontology (GO) molecular function analysis (Figure S3) showing upregulated genes related to kinase function, and down regulated genes related to ribosomal activity and function, both of which are known to be regulated by Ca^2+^ [49].

Changes in expression of genes linked to the pro-regenerative and anti-inflammatory effects of hMSCs were identified, summarised in Table 3. TGF-β1 was significantly upregulated in both CU-701 syringe and CU-701 spray vs. PBS syringe contrasts. TGF-β1 has been implicated in the recruitment of stem/progenitor cells for tissue regeneration [50], though further investigation would be needed to determine whether pectin causes this effect with MSCs. There was no significant change in the expression of the other genes searched for, indicating the pectin or spraying process does not affect the expression of these factors important in the pro-regenerative effects of hMSCs.

**Table 3:** Factors implicated in the pro-regenerative and anti-inflammatory effects of hMSCs

Secreted Factors	Function
Growth Factors	Stimulate cell proliferation, angiogenesis, and tissue repair. Examples include TGF-β, VEGF, FGF, PDGF, IGF, and HGF [4]
Anti-inflammatory Molecules	Suppress inflammation and modulate immune response. Examples include IL-10, IDO, and PGE2 [38]
Extracellular Vesicles	Deliver therapeutic cargo, promote tissue repair, and modulate immune responses [51]
Immunomodulatory Factors	Modulate immune cell activity, suppress inflammation, and promote tissue repair. Examples include NO, IL-6, and galectins [38]
Neurotrophic Factors	Support neuronal survival, growth, and differentiation. Examples include BDNF, NGF, and GDNF [37]

### Limitations

Early biocompatibility testing of the pectin showed the higher Ca^2+^ concentration trended towards higher cell viability, therefore was selected for the further in vitro characterisation. The pectin also formed more stable gels at using 100 mM CaCl_2_ and was therefore a suitable concentration to use in vitro. However, the later differential gene expression studies highlighted the problems with using high calcium concentrations. Translating these in vitro studies to in vivo studies to test efficacy of the cell therapy would require a reduction in the concentration of Ca^2+^ used. In addition to gelation by Ca^2+^, LM pectin demonstrates strong mucoadhesive properties [52]. This property should reduce the concentration of Ca^2+^ required to form a gel; future investigations should aim to bridge the gap between this in vitro work, and translation to in vivo animal studies with respect to the Ca^2+^ used. Additionally, translation in vivo will address the longer-term viability and efficacy of the sprayed cell therapy beyond what was tested in this study. A 24-hour time point was selected in this study to understand the immediate effects of the spraying process, and the process of spraying the hMSCs within the pectin solution. However, in vivo the hMSCs will be affected by the damaged environment; whether the pectin protects the cells from the damage post-delivery and therefore enhances therapeutic benefit will be addressed in future studies.

## Conclusions

Overall, the results of this study show that the syringe-based spray device is suitable for the delivery of hMSCs. Viability of the hMSCs was not affected by the spraying process, and the transcriptome of the cells after spraying is not altered when compared to cells not subjected to the spraying process. Pectin has been demonstrated to be biocompatible, and suitable as a material for use in the spraying process. The calcium used in the gelation of the pectin appears to have affected the transcriptome of the cells, however the concentration of calcium used would be reduced when moving on to ex vivo and in vivo studies, due to the mucoadhesive properties of pectin.

## Electronic supplementary material

Below is the link to the electronic supplementary material.

Supplementary Material 1

Supplementary Material 2

Supplementary Material 3

Supplementary Material 4

Supplementary Material 5

## Data Availability

Data related to this study are provided in this article and the supplementary information. The RNA sequencing raw data is available from Nottingham Research Repository DOI: 10.17639/nott.7505 or from the corresponding author upon request.

## Abbreviations

DM: Degree of methylation
DA: Degree of amidation
hMSCs: Human mesenchymal stem cells
LM: Low-methyl
LDH: Lactate dehydrogenase
MSCs: Mesenchymal stem cells
