# Supplementary material for: An evaluation of spraying as a delivery method for human mesenchymal stem cells suspended in low-methyl pectin solutions
Source: Stem Cell Res Ther. 2025 May 16;16:246. doi: 10.1186/s13287-025-04331-4 (PMC12085057; doi:10.1186/s13287-025-04331-4)
Supplement: Supplementary file 1 — Supplementary Material 1 [file 13287_2025_4331_MOESM1_ESM.docx]

Figure S1 – Viscosity of 1% w/v purified pectin solutions made in PBS. Viscosity measurements were carried out using a cone-and-plate instrument set up. 25 measurements were taken logarithmically from 100 − 10,000 1/s, with measurement times ranging from 1–25 s with a logarithmic ramp. the plate was set to 25°C. Error bars show the standard deviation, n = 3.
