# Supplementary material for: An evaluation of spraying as a delivery method for human mesenchymal stem cells suspended in low-methyl pectin solutions
Source: Stem Cell Res Ther. 2025 May 16;16:246. doi: 10.1186/s13287-025-04331-4 (PMC12085057; doi:10.1186/s13287-025-04331-4)
Supplement: Supplementary file 2 — Supplementary Material 2 [file 13287_2025_4331_MOESM2_ESM.docx]

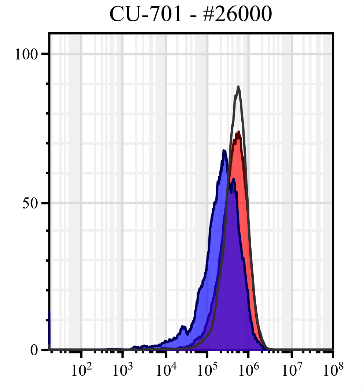

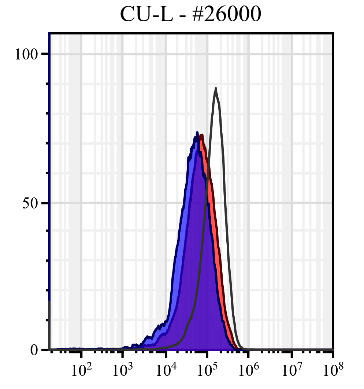

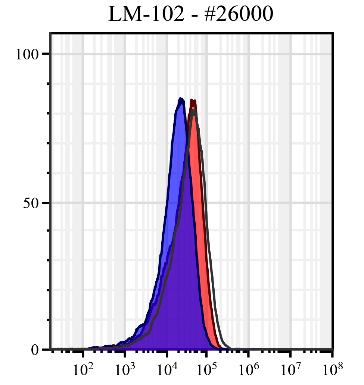

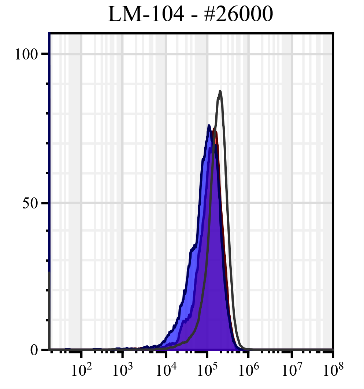

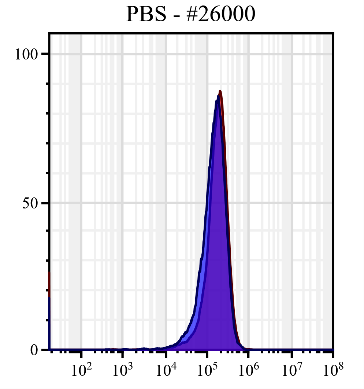

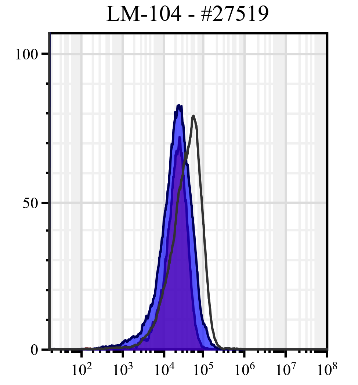

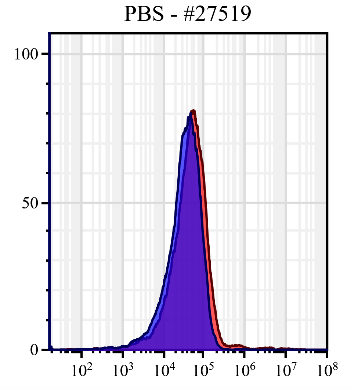

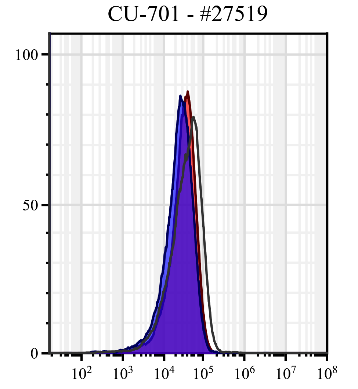

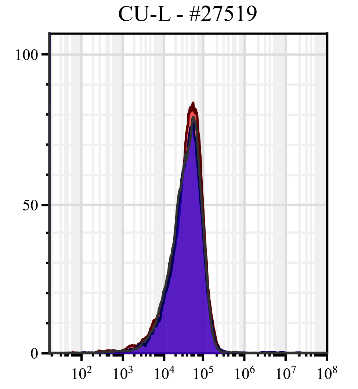

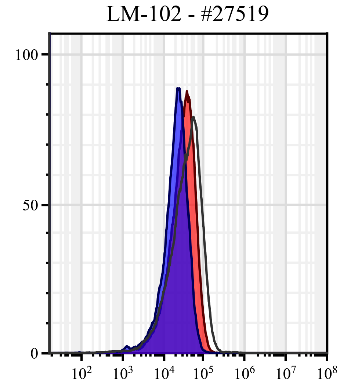

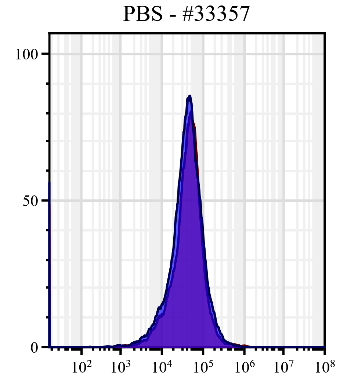

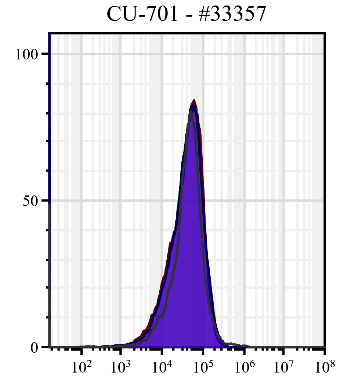

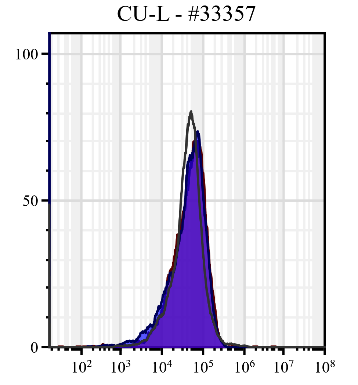

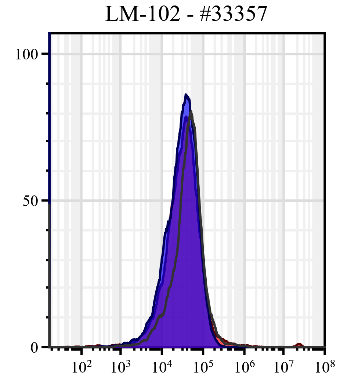

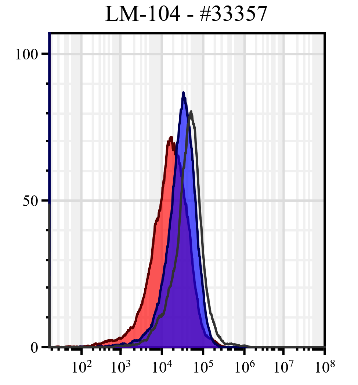


Donor #26000

Donor #27519

Donor #33357

PBS

CU-701

LM-102

LM-104

CU-L

Figure S2 – CD105 PerCP-Cy5.5™ flow cytometry overlay analysis showing the histogram plots for each donor and pectin condition. hMSCs (500 µL at 100,000 cells/mL) were added to 24-well plates by syringing or spraying (mucosal atomisation device sprayer) in 1xPBS or 1% w/v pectin. 50 µL of 100 mM CaCl_2_ were added to wells before cells to induce gelation. Samples were incubated for 24-hours before being extracted from pectin gels and removed from wells to form a single cell suspension. Histograms show cells from gates on the forward scatter-area (FSC-A) vs side scatter-area (SSC-A) plot to select for cells and disregard debris, then from gates set on the SSC-A vs SSC-H plot to select for single cell events. Black outline = PBS control, red = syringed samples, blue = sprayed samples.
