# Supplementary material for: An evaluation of spraying as a delivery method for human mesenchymal stem cells suspended in low-methyl pectin solutions
Source: Stem Cell Res Ther. 2025 May 16;16:246. doi: 10.1186/s13287-025-04331-4 (PMC12085057; doi:10.1186/s13287-025-04331-4)
Supplement: Supplementary file 3 — Supplementary Material 3 [file 13287_2025_4331_MOESM3_ESM.docx]

Figure S3 – Gene ontology (GO) analysis of differentially expressed genes for the CU-701 spray delivery hMSCs vs the PBS syringe delivery hMSCs comparison, for biological function, cellular components, and molecular function.
