# Supplementary material for: An evaluation of spraying as a delivery method for human mesenchymal stem cells suspended in low-methyl pectin solutions
Source: Stem Cell Res Ther. 2025 May 16;16:246. doi: 10.1186/s13287-025-04331-4 (PMC12085057; doi:10.1186/s13287-025-04331-4)
Supplement: Supplementary file 4 — Supplementary Material 4 [file 13287_2025_4331_MOESM4_ESM.docx]

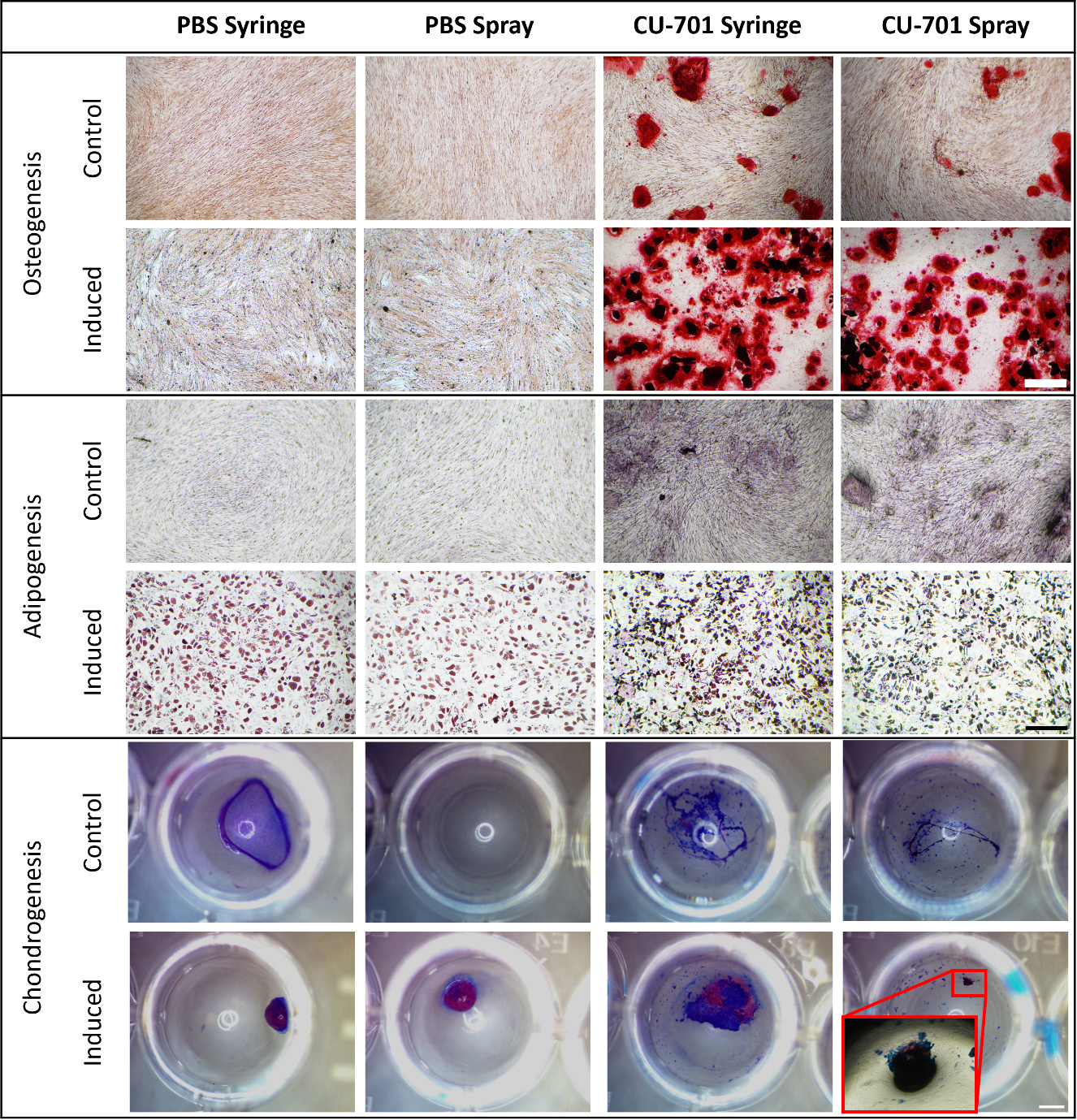


Figure S4 - Osteogenic, adipogenic, and chondrogenic differentiation analysis. Representative images show Alizarin Red S staining appeared to be absorbed by pectin fragments resulting in bright red interference. Oil Red O staining was observed in all conditions cultured in adipogenic medium, with no staining in conditions cultured in basal growth media (control); cells were counterstained with haematoxylin as reference. Chondrogenesis was observed by both morphological alterations and Alcian blue staining (blue), nuclear fast red (red) was used as reference. Scale bars in both osteogenesis and adipogenesis represent 200 µm; in chondrogenesis represents 1 mm.
