# Supplementary material for: An evaluation of spraying as a delivery method for human mesenchymal stem cells suspended in low-methyl pectin solutions
Source: Stem Cell Res Ther. 2025 May 16;16:246. doi: 10.1186/s13287-025-04331-4 (PMC12085057; doi:10.1186/s13287-025-04331-4)
Supplement: Supplementary file 5 — Supplementary Material 5 [file 13287_2025_4331_MOESM5_ESM.docx]

**Supplementary methods for gene ontology analysis**

GO & KEGG Pathway Analysis Significantly, differentially expressed genes (*p* < 0.05) with a baseMean value of > 50 and log^2 fold change of +/- > 0.585 (equivalent to a 1.5x change) were selected for GO analysis. The ‘enrichGO’ function of the R package ‘clusterProfiler’ (Yu et al., 2012; Wu et al., 2021) was used to assign enrichment GO categories for each set of genes, using the R package ‘org.Hs.eg.db’ (Carlson, 2019) as the source of annotations. Biological process, molecular function and cellular component sub-ontologies were calculated for each set. Gene lists also underwent a KEGG enrichment analysis, again using ‘clusterProfiler’.

**Supplementary method for differentiation**

MSC trilineage differentiation was induced using Lonza osteogenic, chondrogenic and adipogenic differentiation BulletKits (PT-3002, PT-3003, PT-3004, respectively). MSCs were suspended at 1x10^5^ cells/mL of pectin solution or PBS as negative control. These were seeded in a 12 well plate with basal media for 24h, before being removed and reseeded at 2x10^5^ cells/well in 24 well plates. These were allowed to adhere for 48h, followed by chemically induction of differentiation for 14 days for osteogenesis and adipogenesis; 21 days for chondrogenesis. Upon completion of differentiation, MSCs were fixed with 4% formaldehyde for 30 min followed by staining with differentiation markers. Osteogenic differentiation was observed by staining with 1% (w/v) Alizarin Red S aqueous solution, pH 4.1, for 30 min followed by complete removal of residual staining solution in large amounts of deionised water. Adipogenic differentiation was observed by staining with 0.21% (w/v) Oil Red O solution in 60% isopropanol for 10 min followed by complete removal of residual staining solution in large amounts of deionised water. MSCs were then counterstained with haematoxylin for 5 min then rinsed with Scott’s water substitute. Chondrogenic differentiation was observed by staining with 1% (w/v) Alcian blue 8GX aqueous solution containing 3% acetic acid for 30 minutes at 37°C. samples were washed with 3% acetic acid followed by water. Samples were counterstained with nuclear fast red solution for 5 min. Images were acquired using Nikon ECLIPSE Ts2 microscope or AmScope LED-144S stereo microscope equipped with MU1000 fine-coloured camera.

CARLSON, M. 2019. org.Hs.eg.db: Genome wide annotation for Human*. R package version 3.8.2.*

WU, T., HU, E., XU, S., CHEN, M., GUO, P., DAI, Z., FENG, T., ZHOU, L., TANG, W., ZHAN, L., FU, Z. & LU, X. 2021. ClusterProfiler 4.0: A universal enrichment tool for interpreting omics data. *The Innovation,* 2, 3.

YU, G., WANG, L. G., HAN, Y. & HE, Q. Y. 2012. ClusterProfiler: an R package for comparing biological themes among gene clusters. *OMICS A Journal of Integrative Biology*, 16, 5.
